# Autophagy Deficiency Leads to Impaired Antioxidant Defense via p62-FOXO1/3 Axis

**DOI:** 10.1155/2019/2526314

**Published:** 2019-12-17

**Authors:** Lin Zhao, Hao Li, Yan Wang, Adi Zheng, Liu Cao, Jiankang Liu

**Affiliations:** ^1^Institute of Mitochondrial Biology and Medicine, The Key Laboratory of Biomedical Information Engineering of Ministry of Education, School of Life Science and Technology and Frontier Institute of Science and Technology, Xi'an Jiaotong University, Xi'an 710049, China; ^2^Key Laboratory of Medical Cell Biology, China Medical University, Shenyang 110001, China

## Abstract

Autophagy, an intracellular degradation mechanism eliminating unused or damaged cytoplasmic components for recycling, is often activated in response to diverse types of stress, profoundly influencing cellular physiology or pathophysiology. Upon encountering oxidative stress, autophagy acts rapidly and effectively to remove oxidized proteins or organelles, including damaged mitochondria that generate more ROS, thereby indirectly contributing to the maintenance of redox homeostasis. Emerging studies are shedding light on the crosstalks among autophagy, mitochondria, and oxidative stress; however, whether and how autophagy could directly modulate antioxidant defense and redox homeostasis remains unaddressed. Here, we showed mitochondrial dysfunction, elevated ROS level, impaired antioxidant enzymes, and loss of FOXO1/3 in autophagy deficiency cellular models established by either chemical inhibitors or knocking down/out key molecules implementing autophagy, and overexpression of FOXO1/3 restored antioxidant enzymes hence suppressed elevated ROS; knockdown of p62 increased protein level of FOXO1/3 and recovered FOXO1 in Atg5-knockdown cells. Our data demonstrates that the loss of FOXO1/3 is responsible for the impairment of antioxidant enzymes and the consequent elevation of ROS, and accumulation of p62 under condition of autophagy deficiency might be mediating the loss of FOXO1/3. Furthermore, we found in an animal model that the p62-FOXO1/3 axis could be dominant in aging liver but not in type 2 diabetic liver. Together, these evidences uncover the p62-FOXO1/3 axis as the molecular cue that underlies the impairment of antioxidant defense in autophagy deficiency and suggest its potential involvement in aging, substantiating the impact of inadequate autophagy on mitochondria and redox homeostasis.

## 1. Introduction

Autophagy is an intrinsic process that disassembles and degrades unused or damaged cellular components including organelles like mitochondria, macromolecules like proteins or lipids, and other cytoplasmic materials. In contrast to the other two defined types of autophagy, microautophagy and chaperone-mediated autophagy, macroautophagy (hereafter referred to as autophagy) is a highly regulated process characterized by the formation of the intermediary autophagosome that later fuses with the lysosome to deliver cytoplasmic cargo, and it is the one getting intensive attention in the past two decades [1–3]. A cohort of ATG proteins composing autophagy machinery and the mechanisms of the four major steps of autophagy have been characterized in detail from yeasts to the mammalian system [4], and the quest for the diverse cellular roles of autophagy and the complex impact of the deregulated autophagy pathway on health and disease, as well as the potential of therapeutically manipulating autophagy, both induction and inhibition, in clinical applications is still ongoing [5–12].

Autophagy, with an essential role in homeostasis and normal physiology, has been linked with longevity, aging [13], and multiple age-related diseases like neurodegenerative disorders, cancer, cardiovascular disease, and metabolic diseases [10, 13–15], and emerging data suggest that most components of the molecular machinery for autophagy have autophagy-independent roles [16]. However, the relation between autophagy and diseases remains elusive. Autophagy is often recognized as a double-edged sword having competing or opposing effects even in the same pathophysiological scenario, and only with better understanding of the detailed molecular mechanisms in play can we develop worthwhile translational and clinical studies [17].

Meanwhile, the progressive accumulation of dysfunctional mitochondria and oxidative damage is widely recognized to play a causal role in aging and in a wide variety of age-associated diseases according to the mitochondrial free-radical theory of aging [18], which was prevalent for more than half a century and developed into the redox theory of aging recently [19]. Indeed, major causes of human morbidity and mortality are associated with oxidative stress, which occurs with a high amount of oxidants and ineffective antioxidant defense, leading to a disruption of a repertoire of redox signalings and consequently impacting fundamental cellular activities like bioenergetics, formation of metabolite and macromolecule structure, and spatial and temporal activation/deactivation of protein switches, eventually expediting cellular senescence and death [19]. It should be pointed out that antioxidant enzymes fulfill a major role in antioxidant defense rather than small-molecule antioxidant compounds in the endeavor of maintaining redox balance [20].

There exists an intricate crosstalk between autophagy and oxidative stress, intimately involving mitochondria and redox signaling ([21, 22]). Oxidative stress acts as the converging point of various types of stimuli for autophagy, with reactive oxygen species (ROS) being an important signal transducer mediated by macromolecule damage and reversible modifications of thiol-containing proteins [23]. ROS activate autophagy flux ([24]), and ROS elimination by antioxidant treatment or overexpression of antioxidant enzymes has been shown to inhibit autophagy in many models ([25, 26]). On the other side of the autophagy-oxidative stress interconnection, evidences are also emerging. Autophagic clearance of diverse damaged molecules may serve as an essential cellular antioxidant pathway [14]; autophagy-deficient models exhibit oxidative stress, probably due to the accumulation of dysfunctional mitochondria and the consequent increase of ROS generation [27–29]. Nevertheless, besides clearing oxidized cellular components and protecting mitochondrial state for less generation of ROS at source, whether autophagy affects redox homeostasis by regulating antioxidant enzymes, the key player in maintaining redox balance, arises as a key question to be addressed; if so, the mediating molecular mechanisms also need to be elucidated.

It has been reported that p62 could sequester Kelch-like ECH-associated protein 1 (Keap1) for degradation via autophagy and detach nuclear factor erythroid 2-related factor 2(Nrf2) from Keap1, leading to nuclear translocation of Nrf2 and the subsequent transcriptional activation of antioxidant and detoxifying genes ([23, 30–32]). The Forkhead Box O family of transcription factors (FOXOs) serve as the central regulator of cellular homeostasis encompassing cell cycle arrest, cell death, stress resistance, and cellular metabolism ([33–35]). FOXOs not only participate in the regulation of the transcription of antioxidant enzymes such as catalase, Cu-ZnSOD, and MnSOD [36–38], but also control the process of autophagy by modulating the transcription of autophagy-related genes such as LC3, Gabarapl1, Atg12, Vps34, Bnip3, and Bnip3l [39–41] or by directly interacting with Atg7 to regulate the induction of autophagy ([42]). Besides the wide range of downstream effects, FOXOs' activities are under tight control at multiple levels by diverse upstream regulators and have been recently proposed to be signaling integrators coordinating cellular homeostasis and response to environmental changes over time; redox status modulates FOXOs directly by reversible Cys oxidation on FOXOs or indirectly by affecting FOXO regulators [33]. However, whether autophagy is among the many upstream regulators for FOXOs or whether autophagy can conversely regulate FOXOs and exert influence on cellular homeostasis especially redox homeostasis remains uninvestigated.

In the current study, we reported that the expression of FOXO1/3 transcription factors as well as antioxidant enzymes including Cu-ZnSOD, MnSOD, and catalase significantly decreased in response to the inhibition of autophagy and loss of FOXO1/3 caused by accumulated p62 was responsible for the impairment of the antioxidant system; hence, we propose that the p62-FOXO1/3 axis is the underlying pathway by which antioxidant defense is impaired in the condition of autophagy deficiency. Furthermore, observations in animal liver tissue imply the involvement of this regulatory axis in aging but not in a type 2 diabetic context. Our results further strengthened p62 as an autophagy-related signaling molecule. Our data, which uncovers the p62-FOXO1/3 axis, contribute to the depiction of a more comprehensive molecular basis by which p62 downregulates the antioxidant defense system in addition to the already reported p62/keap1/Nrf2 pathway which upregulates the antioxidant defense system. These findings promote our understanding of the modulation of the expression of antioxidant enzymes mediated by p62 and FOXO1/3, help decode the role of autophagy and redox homeostasis in diseases, and hint towards potential therapeutic opportunities related to the p62-FOXO1/3 axis.

## 2. Materials and Methods

### 2.1. Cell Culture and Chemicals

Wild-type (WT) and Atg7-deficient (Atg7-/-) MEFs (mouse embryonic fibroblasts) have been described ([29, 43]) and were maintained in H-DMEM (Gibco) supplemented with 15% fetal bovine serum (Biological Industries), 100 U/mL penicillin, and 100 g/mL streptomycin (Sigma-Aldrich). HEK293T cells were purchased from the American Type Culture Collection and maintained in H-DMEM supplemented with 10% fetal bovine serum. Cells were maintained at 37°C in a humidified atmosphere of 5% CO_2_ and 95% air. All plastic ware was from Corning Life Sciences. The inhibitors N-acetyl-L-cysteine (Klotz et al., 2015), chloroquine diphosphate salt (CQ), bafilomycin A1 (bafi A1), 3-methyladenine (3-MA), and other reagents were bought from Sigma-Aldrich and prepared as stock solutions and stored at -20°C until use.

### 2.2. Animals

Sprague-Dawley (SD) male rats were purchased from a commercial breeder (SLAC, Shanghai, China). The rats were housed in temperature- (24-26°C) and humidity- (60%) controlled animal rooms and maintained on a 12 h light/12 h dark cycle (light on from 08:00 a.m. to 08:00 p.m.) with free access to food and water throughout the experiments. Four-week-old male rats weighing 180–200 g were used to start the experiments, and after reaching 25 months and 5 months of age (old and young groups, respectively), the animals were sacrificed and then liver samples were collected. The C57 mice, B6-OB/Nju (B6/JNju-Lep^em1Cd25^/Nju) mice, and BKS-*db* (BSK-Lepr^em2Cd479^/Nju) mice at 18 weeks of age were provided by the Model Animal Research Center of Nanjing University. The animals were sacrificed after adaptation to the environment, and then liver samples were collected. All of the procedures were performed in accordance with the United States Public Health Services Guide for the Care and Use of Laboratory Animals, and all efforts were made to minimize the suffering and the number of animals used in this study.

### 2.3. Transfection

siRNA targeting Atg5, Beclin1, and p62 were purchased from GenePharma (Shanghai, China) and were transfected into HEK293T cells using Lipofectamine 2000 (Invitrogen) with Opti-MEM® I Reduced Serum Medium (Gibco) according to the manufacturer's instructions. The siRNA sequences could be found in [Supplementary-material supplementary-material-1]. pcDNA3-Flag-FOXO1 and GFP-FOXO3 constructs were kind gifts from Dr. Kun-Liang Guan (University of California, San Diego) and Dr. Mien-Chie Hung (MD Anderson Cancer Center), respectively, and have been previously described [44, 45]. The plasmids were transfected into HEK293T cells with empty vectors as the negative control using X-tremeGENE HP DNA Transfection Reagent (Roche) according to the manufacturer's instructions.

### 2.4. Western Blotting

Cells were lysed and centrifuged at 13,000 g for 15 min at 4°C. The supernatants were collected, and protein concentrations were determined with the BCA Protein Assay Kit (Pierce). Equal amounts of protein samples were applied to sodium dodecyl sulfate polyacrylamide gel electrophoresis (SDS-PAGE) gels, transferred to pure nitrocellulose membranes (PerkinElmer), and blocked with 5% nonfat milk for 2 hours. The membranes were then incubated with the indicated primary antibodies at 4°C overnight. Primary antibodies used in this study were *β*-actin from Sigma-Aldrich; Atg7, Atg5, Beclin1, and FOXO1 from Cell Signaling Technology; FOXO3 from Sigma-Aldrich; p62, Cu-ZnSOD, MnSOD, and catalase from Santa Cruz Biotechnology; Complex I Ndufs3, Complex I Ndufa9, Complex II subunit 30 kDa Ip, Complex III subunit Core 1, Complex IV Subunit I, and ATP Synthase Subunit Alpha from Invitrogen; and LC3B from Abcam. The membranes were incubated with secondary peroxidase-conjugated antibodies (Jackson ImmunoResearch) at room temperature for 1 h. Chemiluminescent detection was performed by ECL (Pierce). The results were analyzed and quantified by Quantity One Software (Bio-Rad) to obtain the optical density ratio of the target protein to *β*-actin. One representative result was shown from at least three independent experiments.

### 2.5. Quantitative RT-PCR

Total RNA was extracted from cells using Tripure (Roche) according to the manufacturer's protocol. Reverse transcription was performed using the PrimeScript RT-PCR Kit (TaKaRa) followed by semiquantitative real-time PCR with specific primers described in [Supplementary-material supplementary-material-1] using SYBR Premix Ex Taq (TaKaRa). Real-time PCR was performed on a real-time PCR system (Eppendorf, Germany) with an initial step of 10 min at 95°C, followed by 40 cycles of 30 s denaturation at 95°C, 30 s annealing at 60°C, and 20 s extension at 72°C. Melting curves were assessed over the range 60-99°C to ensure specific DNA amplification. Target gene expression was normalized to *β*-actin expression and is shown as levels relative to control samples.

### 2.6. Activity Assays for Mitochondrial Electron Transport Chain Complexes

Activities of NADH-ubiquinone oxidoreductase (Complex I) and succinate-CoQ oxidoreductase (Complex II) were measured spectrometrically using conventional assays as described [46, 47].

### 2.7. Assays for Mitochondrial Membrane Potential and ROS Generation

Mitochondrial membrane potential was assessed in cells using a mitochondria-specific cationic probe JC-1 (Invitrogen). Intracellular ROS levels were determined by using of DCFDA. The assays have been described [48].

### 2.8. Total DNA Isolation and mtDNA Copy Number Detection

Total DNA was extracted using the QIAamp DNA Mini Kit (Qiagen), and quantitative PCR was performed using mitochondrial DNA and genomic DNA-specific primers described in [Supplementary-material supplementary-material-1]. Final results were expressed as changes relative to control samples in mitochondrial D-loop levels relative to the 18S rRNA gene.

### 2.9. Assay for Oxygen Consumption Capacity

Cellular mitochondrial respiration rates (oxygen consumption rates (OCR)) of certain numbers of cells (30,000 for 293T and 20,000 for MEFs) were investigated using the Seahorse Extracellular Flux Analyzer (Seahorse Bioscience, North Billerica, MA) according to the manufacturer's instructions ([49]). Resulting rates were adjusted to the number of cells per well after detection.

### 2.10. Intracellular Superoxide Dismutase and Catalase Activity Measurements

Superoxide dismutase and catalase activity were measured using commercially available kits (Jiancheng Biochemical Research, Inc.) following the instructions provided by the manufacturer. The activities of antioxidant enzymes were expressed as changes relative to control samples.

### 2.11. Cell Viability

MEFs were plated in 24-well plates and incubated overnight. Cells were treated with 1 mM tBHP (*tert*-butyl hydroperoxide) for the indicated time periods and then incubated with 0.5 mg/mL MTT (3-(4,5-dimethylthiazol-2-yl)-2,5-diphenyltetrazolium bromide) medium. The optical density was determined by a microplate spectrophotometer at a wavelength of 550 nm.

### 2.12. Protein Carbonylation Assay

Protein carbonyls in soluble proteins were assayed using the OxyBlot protein oxidation detection kit (Cell Biolabs, USA). Protein carbonyls were labeled with 2,4-dinitrophenylhydrazine and detected by Western blot. As a negative loading control, equal amounts of samples were subjected to 10% SDS-PAGE and stained with Coomassie brilliant blue.

### 2.13. Statistical Analysis

All data are expressed as the means ± SEM. Immunoblots are representative results of at least three independent experiments. Statistical significance was analyzed by unpaired two-tailed Student's *t*-test or ANOVA. A *p* value of less than 0.05 was considered statistically significant.

## 3. Results

### 3.1. Mitochondrial Dysfunction, Increased ROS, and Impairment of Antioxidant Enzymes Occur in Response to Chemical Inhibitors of Autophagy or under Circumstance of Atg7 Knockout or Knockdown of Other Autophagy Components

**Figure 1:**
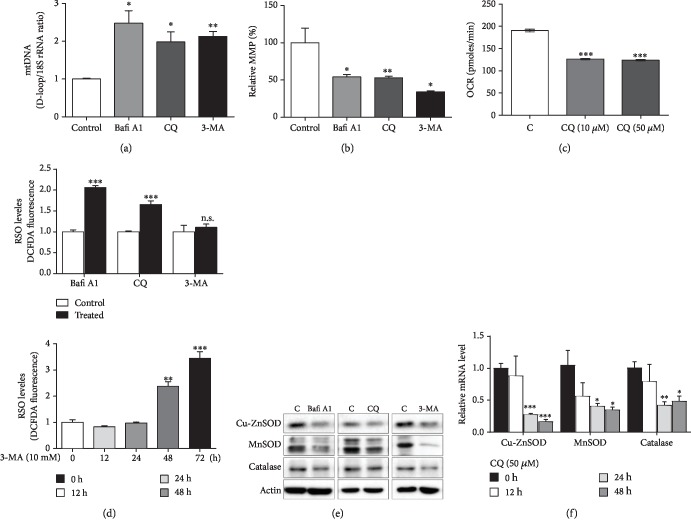
Mitochondrial dysfunction, increased ROS, and impairment of antioxidant enzymes occur in response to chemical inhibitors of autophagy. (a, b, d, and e) HEK293T cells were treated with autophagy inhibitors Bafi A1 (100 nM), CQ (50 *μ*M), or 3-MA (10 mM) for 24 hours or with 10 mM 3-MA in a time course within 72 hours before harvest. Then, mitochondrial DNA (as D-loop DNA) copy number was detected by real-time PCR (*n* = 3) (a), mitochondrial membrane potential (MMP) was detected with the JC-1 fluorescent probe (*n* > 9) (b), ROS level alterations were determined by a fluorescence microplate reader after staining with DCFDA (*n* = 3) (d), and protein levels of antioxidant enzymes were determined by W.B. (e). (c and f) WT MEF cells were treated with CQ at indicated concentrations for 24 hours or with 50 *μ*M CQ for the indicated time period before harvest, then oxygen consumption rate (OCR) was determined using the Seahorse XF24 Analyzer (*n* = 6‐7) (c) and mRNA levels of antioxidant enzymes were determined by q-RT-PCR (*n* = 3) (f). Values are represented as mean ± SEM. ^∗^*p* < 0.05; ^∗∗^*p* < 0.01; ^∗∗∗^*p* < 0.001; ^∗^ vs. control group.

To determine the impact of autophagy inhibition on cellular mitochondrial homeostasis and redox balance, we first measured mitochondrial alterations and ROS level in HEK293T cells or MEF cells treated with three different chemical inhibitors of autophagy, Bafi A1 (bafilomycin A1, a widely used inhibitor disrupting autophagosome-lysosome fusion), CQ (chloroquine diphosphate salt, reported to inhibit autophagy), and 3-MA (3-methyladenine, a selective PI3K inhibitor that also blocks autophagosome formation). CQ treatment results in a time-responsive decline in mRNA levels of key autophagy components Atg12, Bnip3, and LC3, indicating the inhibition of autophagy (Figures [Supplementary-material supplementary-material-1]). All three inhibitors induced a more than a two-fold increase in mitochondrial DNA copy number (Figure 1(a)) as well as a robust decrease to less than one half in mitochondrial membrane potential (MMP) (Figure 1(b)) in HEK293T cells, and CQ significantly reduced oxygen consumption rate (OCR) in MEF cells (Figure 1(c)). These data indicate that inhibition of autophagy results in increased mitochondrial number but impaired mitochondrial function, which make sense as damaged mitochondria accumulate in the cell due to a lack of degradation through mitophagy, an important mitochondrial quality control mechanism. Concurrently, these inhibitors also increased ROS level significantly and 3-MA was found to boost ROS in a time-dependent manner, especially with prolonged time (Figure 1(d)). Since ROS generated in the mitochondria as a byproduct of the electron transport chain accounts for the majority of cellular ROS, accumulated dysfunctional mitochondria would release more ROS and eventually enhance intracellular oxidative stress if the endogenous antioxidant defense remains poor. We then went on to check the expression of the most important antioxidant enzymes Cu-ZnSOD, MnSOD, and catalase and detected a significant decrease in their protein levels when cells were treated with any of the three different autophagy inhibitors (Figure 1(e)), and the reduction of their mRNA levels by CQ treatment exhibited time-dependence (Figure 1(f)). So, inhibition of autophagy can trigger oxidative stress by paralyzing mitochondria to generate more ROS on the one hand, on the other sabotaging endogenous antioxidant defense.

**Figure 2:**
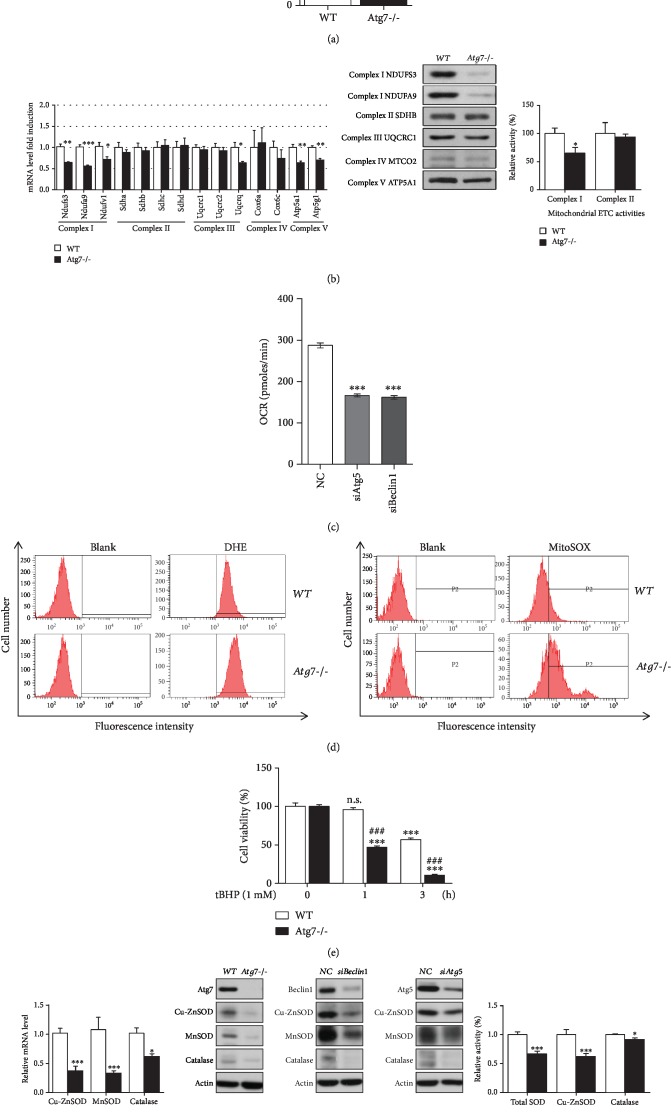
Mitochondrial dysfunction, increased ROS, decreased capacity to survive oxidative stress, and impairment of antioxidant enzymes also occur when Atg7 is deleted or other autophagy components are knocked down by siRNA. (a) Mitochondrial membrane potential (MMP) in WT and Atg7-/- MEF cells (*n* > 9) was detected with the JC-1 probe. (b) Relative mRNA levels of subunits of mitochondrial respiratory complexes were determined by quantitative RT-PCR (*n* = 4), relative protein levels of subunits of mitochondrial respiratory complexes were determined by W.B., and relative activities of mitochondrial respiratory complexes I and II were measured in isolated mitochondria from WT and Atg7-/- MEF cells (*n=8*). (c) HEK293T cells were transfected with negative control siRNA, siAtg5, or siBeclin1, then OCR was determined using the Seahorse XF24 Analyzer (*n* = 6‐7). (d) ROS level changes and mitochondrial superoxide level changes in WT and Atg7-/- MEF cells were detected by flow cytometry using 5 *μ*M DHE or 5 *μ*M MitoSOX Red Mitochondrial Superoxide Indicator. (e) WT and Atg7-/- MEFs were treated with 1 mM tBHP for the indicated time period, and their cell viability was determined by MTT (*n* = 10). (f) mRNA levels of Cu-ZnSOD, MnSOD, and catalase in WT and Atg7-/- MEF cells were determined by q-RT-PCR (*n* = 6); protein levels of antioxidant enzymes in Atg7-/- MEF cells in comparison to WT or siBeclin1- or siAtg5-transfected HEK293T cells in comparison to negative control siRNA were determined by W.B.; relative enzymatic activities of antioxidant enzymes were measured in WT and Atg7-/- MEF cells (*n* = 6). Values are represented as mean ± SEM. ^∗^*p* < 0.05; ^∗∗^*p* < 0.01; ^∗∗∗^^,###^*p* < 0.001; ^∗^ vs. control group or WT MEF cells, ^#^ vs. WT MEF cells with the same treatment time period.

We further corroborated our findings employing Atg7-/- and WT MEF cells. The mRNA levels of key autophagy components Atg5, Atg12, Bnip3, gaparapl1, LC3, and vps34 were universally decreased in Atg7-/- MEF cells compared with WT ([Supplementary-material supplementary-material-1]); the protein level of Atg5 was decreased as much as Atg7; LC3II was almost absent; and p62 was increased in Atg7-/- MEF cells ([Supplementary-material supplementary-material-1]), indicating an obvious state of autophagy deficiency in Atg7-/- MEF cells. MMP was significantly lowered in Atg7-/- MEF cells than WT (Figure 2(a)). With deeper investigation, we found that several subunits of the mitochondrial electron transport chain (ETC) complexes exhibited a substantial decrease in both mRNA transcript and protein levels, especially subunits of Complexes I and V, and the activity of ETC Complex I dropped significantly in Atg7-/- MEF cells compared with WT MEF cells (Figure 2(b)). In addition, we used siRNA to knockdown other components of autophagy, Atg5 or Beclin1 in particular, to exclude the specific effect of Atg7, and as expected OCR dropped substantially in HEK293T cells (Figure 2(c)). Clearly, these findings verify the adverse impact of autophagy deficiency on mitochondrial function. Since the mitochondrial rate of reactive oxygen species (mitROS) production is recently considered of more importance than overall ROS level, we next examined ROS level with two different fluorescent probes, one indicating for intracellular ROS, the other targeting for mitochondrial superoxide, and both intracellular ROS and mitochondrial superoxide increased in Atg7-/- MEF cells compared with WT MEF cells (Figure 2(d)). Besides, Atg7-/- MEF cells showed higher vulnerability than WT MEF cells when challenged with tBHP (*tert*-butyl hydroperoxide), an exogenous inducer of oxidative stress (Figure 2(e)). Underneath the increased ROS level and higher vulnerability to oxidative stress of Atg7-/- MEF cells, we observed a drastic decrease in both mRNA levels and protein levels of key antioxidant enzymes, Cu-ZnSOD, MnSOD, and catalase; moreover, even their enzymatic activities were found to be universally reduced in Atg7-/- MEF cells compared with WT MEF cells (Figure 2(f)). Likewise, Atg5 or Beclin1 knockdown by siRNA also led to decreased protein levels of all three antioxidant enzymes in HEK293T cells (Figure 2(f)). These data demonstrate that autophagy deficiency can cause the impairment of antioxidant defense. Together, Atg7 knockout or knockdown of other autophagy components simultaneously destroyed mitochondrial homeostasis and endogenous antioxidant defense, which is consistent with the findings with autophagy inhibitors.

From the above results we can see that in general, inhibition of autophagy or autophagy deficiency leads to the accumulation of dysfunctional mitochondria, which release more ROS and in turn cause further damage to one of its major target mitochondria, thus establishing a vicious cycle between mitochondrial malfunction and cellular redox imbalance. Cells possess an innate powerful antioxidant system capable of attenuating intracellular free radicals to protect against their attack, among which an array of antioxidant enzymes play the key role. In condition of autophagy inhibition, mitochondrial dysfunction and the consequent intracellular oxidative stress could only be left exacerbated with the downregulation of antioxidant enzymes and decrease of enzymatic activities, and the lack of mitophagy eliminating damaged mitochondria.

### 3.2. Impairment of Antioxidant Enzymes and Elevation of ROS in Condition of Autophagy Deficiency Is Mediated by the Decrease of FOXO1/3 Transcription Factors

We next sought to investigate the mechanism underlying the downregulation of antioxidant enzymes in condition of autophagy deficiency. Since the amounts of mRNA transcripts of antioxidant enzymes were dramatically reduced in various autophagy deficiency models (Figures 1(f) and 2(f)), we focused on transcriptional regulation and looked into related transcription factors. Nrf2 (nuclear factor erythroid 2-related factor 2), the master regulator of the total antioxidant system or phase II detoxifying enzymes, is a transcription factor that bonds to ARE (Antioxidant Response Element) and activate the expression of cytoprotective enzymes, playing important role in adaptive response to oxidative stress. We found that the protein level of Nrf2 was dramatically higher in Atg7-/- MEF cells than in WT MEF cells ([Supplementary-material supplementary-material-1]), and the expression of NQO1 (NAD (P) H quinone dehydrogenase 1), one of Nrf2's downstream target genes, exhibited higher induction as manifested by higher levels of both mRNA and protein in Atg7-/- MEF cells than in WT MEF cells (Figures [Supplementary-material supplementary-material-1] and [Supplementary-material supplementary-material-1]). The fact that the Nrf2/ARE pathway is activated probably responding to oxidative stress but failing to induce the expression of antioxidant enzymes in the condition of autophagy deficiency suggests the involvement of other players downregulating antioxidant enzymes.

**Figure 3:**
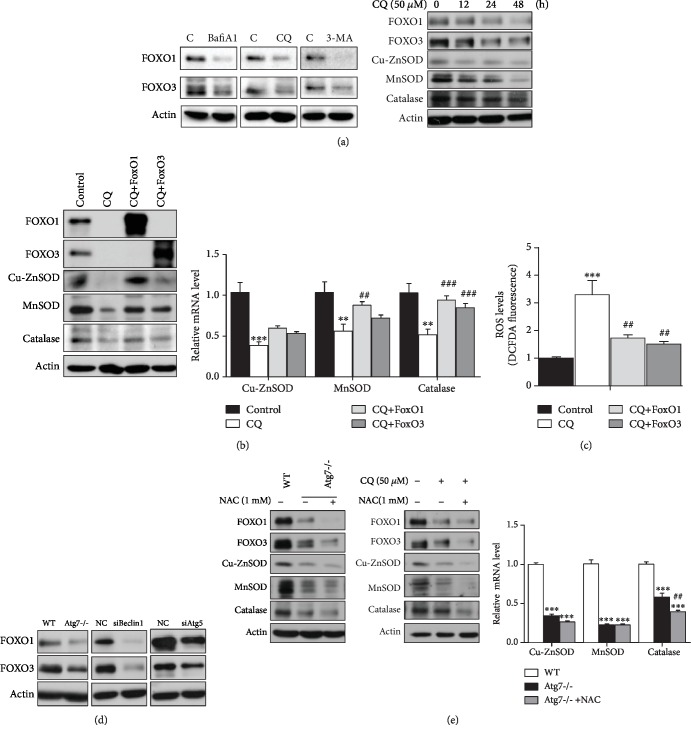
Impairment of antioxidant enzymes and elevation of ROS in a condition of inhibited autophagy is mediated by the decrease of FOXO1/3 transcription factors, and scavenging ROS by NAC not only fails to rescue the loss of FOXOs or antioxidant enzymes but even exacerbates their loss. (a) HEK293T cells were treated with autophagy inhibitors Bafi A1 (100 nM), CQ (50 *μ*M), or 3-MA (10 mM) for 24 h or with 50 *μ*M CQ for 0, 12, 24, or 48 hours before harvest, then protein levels of FOXO transcription factors and antioxidant enzymes were determined by W.B. (b and c) HEK293T cells were treated with 50 *μ*M CQ for 24 hours before transfection with FOXO1 or FOXO3 plasmids, then followed by treatment with 50 *μ*M CQ for another 48 hours before harvest, and then protein and mRNA levels of FOXOs and antioxidant enzymes were relatively determined by W.B. and q-RT-PCR (*n* = 6) (b), and intracellular ROS levels were measured with a DCFDA probe (c) (*n* = 12). (d) Protein levels of FOXO1 and FOXO3 in Atg7-/- MEF cells compared with WT MEF cells, in siAtg5- or siBeclin1-transfected HEK293T cells compared with negative control siRNA transfected ones were determined by W.B. (e) Atg7-/- MEF cells were treated with 1 mM NAC for 48 hours before harvest, HEK293T cells were treated with 1 mM NAC for 48 hours and/or CQ for 24 hours before harvest, then protein levels of FOXOs and antioxidant enzymes were determined by W.B. in both cells, and mRNA levels of antioxidant enzymes were determined by q-RT-PCR (*n* = 6) in MEF cells. Values are represented as mean ± SEM. ^∗^^,#^*p* < 0.05; ^∗∗^^,##^*p* < 0.01; ^∗∗∗^^,###^*p* < 0.001; ^∗^ vs. control group or WT MEF cells, ^#^ vs. treatment with CQ alone or Atg7-/- MEF cells.

We continued to search for the molecules that are held accountable for the decrease of the mRNA levels and the protein levels of antioxidant enzymes in autophagy deficiency models. Because FOXO (Forkhead Box O) transcription factors are also known to regulate the expression of MnSOD, catalase [36], and Cu-ZnSOD [37, 50] through transcriptional control, we examined the protein levels of FOXO1 and FOXO3 in HEK293T cells treated with three different autophagy inhibitors and found both FOXO1 and FOXO3 decreased robustly, and both molecules along with antioxidant enzymes Cu-ZnSOD, MnSOD, and catalase showed a time-dependent decrease in response to a 48-hour time course of CQ treatment (Figure 3(a)). To find out the role that FOXO1/3 plays in impaired antioxidant defense and enhanced oxidative stress in an autophagy deficiency condition, we overexpressed FOXO1 or FOXO3 in CQ-treated HEK293T cells and the recovery of Cu-ZnSOD, MnSOD, and catalase was confirmed in both protein and mRNA levels (Figure 3(b)). Besides, the simultaneous overexpression of FOXO1 and FOXO3 could further induce the increase in mRNA transcripts of antioxidant enzymes ([Supplementary-material supplementary-material-1]). Moreover, as expected, overexpression of FOXO1 or FOXO3 also relieved the intracellular oxidative stress in CQ-treated HEK293T cells, represented by ROS level (Figure 3(c)). We also checked the expression of FOXO1/3 in other autophagy-deficient models. Both protein levels and mRNA levels of FOXO1/3 were dramatically decreased in Atg7-/- MEF cells in comparison to WT MEF cells (Figures [Supplementary-material supplementary-material-1] and [Supplementary-material supplementary-material-1]; Figure 3(d)), and protein levels of both FOXO1 and FOXO3 were substantially decreased in siAtg5- or siBeclin1-transfected HEK293T cells in comparison to negative control siRNA-transfected ones (Figure 3(d)). Collectively, these data verify that it is the downregulation of FOXO1/3 transcription factors by autophagy deficiency that subsequently reduces the transcription of antioxidant enzymes and further exacerbates oxidative stress already existing due to mitochondrial dysfunction induced by inhibition of autophagy.

FOXOs are also considered as an upstream modulator of autophagy; for instance, conditional deletion of FOXOs strongly impairs autophagic flux in adult neurogenesis [51]. This prompted us to examine the impact of FOXO1/3 overexpression on transcription of genes participating in autophagy, and we found that the decreased mRNA levels of Atg12, Bnip3, and LC3 in CQ-treated HEK293T cells were restored by FOXO1/3 overexpression ([Supplementary-material supplementary-material-1]). Therefore, it can be inferred that FOXO1/3 overexpression would alleviate oxidative stress in autophagy-deficient models through simultaneously inducing expression of antioxidant enzymes and restoring expression of genes participating autophagy, while on the other side it can be conjectured that the negative regulation of FOXO1/3 by autophagy deficiency would have exerted additional inhibition on autophagy, forming a feedforward loop resulting in retarded autophagy and the following oxidative stress.

### 3.3. Scavenging ROS by NAC Not Only Fails to Rescue the Loss of FOXOs and Antioxidant Enzymes but Even Exacerbates Their Loss in Autophagy Deficient Condition

Now we know that FOXO1/3 sits at the pivot linking autophagy deficiency to redox imbalance, but what leads to the decrease of FOXO1/3 in a condition of autophagy deficiency emerges as the key question to be addressed. It has already been reported that ROS could modulate not only the transcriptional and posttranscriptional control of FOXO expression but also the activity of FOXO at multiple levels including posttranslational modifications of FOXOs (such as phosphorylation and acetylation), interaction with coregulators, alterations in FOXO subcellular localization, protein synthesis, and stability [52]. As shown in Figure 3(e), NAC (N-acetyl-L-cysteine), a commonly used ROS scavenger, not only failed to recover the loss of FOXO1/3 as well as antioxidant enzymes but even exacerbates their loss in both Atg7-/- MEF cells and CQ-treated HEK293T cells. These findings suggest that the elevation of ROS level in autophagy-deficient cellular models not only made no contribution to the decrease of FOXO1/3 and the impairment of antioxidant enzymes but quite on the contrary had also made its efforts to upregulate or maybe also to activate FOXO1/3 and subsequently to increase the expression of antioxidant enzymes, hence promoting cellular resistance to oxidative stress.

### 3.4. Accumulated p62 under Autophagy Deficiency Condition Is Responsible for the Loss of FOXO1/3

**Figure 4:**
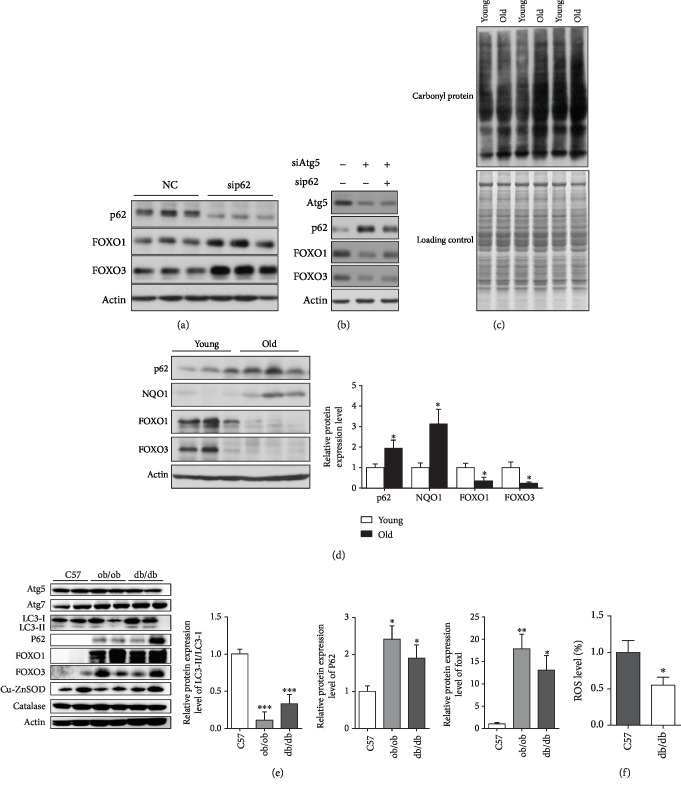
Accumulated p62 in a condition of inhibited autophagy is responsible for the loss of FOXO1/3, which is involved in aging but not dominant in type 2 diabetes mellitus animal models. (a and b) HEK293T cells were transfected with sip62 or negative control siRNA or with sip62 and/or siAtg5 for 48 hours before harvest; indicated protein levels were determined by W.B. (c and d) Liver tissues from SD rats aged five months (young) and 25 months (old) were homogenized to yield lysates, protein carbonylation levels were detected with a commercially available kit, Coomassie brilliant blue staining was used to monitor equal loading control (c), and indicated protein levels were determined by W.B. and densitometry analysis (*n* = 9) (d). (e and f) Liver tissues from 18-week-old ob/ob (B6/JNju-Lep^em1Cd25^/Nju), db/db (BSK-Lepr^em2Cd479^/Nju), and C57 mice were homogenized to yield lysates, indicated protein levels were determined by W.B. and densitometry analysis (*n* = 6) (e), and ROS levels were measured with a DCFDA probe (*n* = 6) (f). Values are represented as mean ± SEM, and statistical analysis were conducted using *t*-test. ^∗^*p* < 0.05; ^∗∗^*p* < 0.01; ^∗∗∗^*p* < 0.001; vs. young or C57.

p62 (p62/SQSTM1 or Sequestosome 1) serves as a scaffold by binding with ubiquitinated cellular cargoes and autophagosomal membrane protein LC3, facilitating substrate degradation of autophagy. Protein aggregates formed by p62 are often used as reporters of retarded autophagy activity. More importantly, it is a multifunctional protein involved in many signal transduction pathways and has been linked to oxidative stress. It prompts us to investigate whether the accumulation of p62 under an autophagy inhibition condition has anything to do with the loss of FOXO1/3. We observed that the protein levels of both FOXO1 and FOXO3 increased when p62 was knocked down by siRNA in HEK293T cells (Figure 4(a)), indicating a negative regulation of FOXO1/3 by p62. More interestingly, knocking down the accumulated p62 in siAtg5-transfected HEK293T cells by sip62 did partially restore the expression of FOXO1/3 (Figure 4(b)), demonstrating that the accumulation of p62 plays an essential role in mediating autophagy deficiency-induced loss of FOXO1/3. Considering the increased p62 protein level and decreased FOXO1/3 mRNA levels in Atg7-/- MEF cells ([Supplementary-material supplementary-material-1]), transcriptional modulation might be involved in the downregulation of FOXO1/3 by p62, though other possibilities could not be ruled out; further studies are needed to elucidate the regulating mechanisms. Altogether, the above results reveal the existence of a negative regulatory axis pointing from p62 to FOXO1/3 and show that autophagy deficiency impairs antioxidant defense via the newly uncovered p62-FOXO1/3 axis. Moreover, these findings also substantiate the existence of a crosstalk between autophagy and FOXO1/3 which is of significance in health and disease.

### 3.5. Decline of Autophagy, Accumulation of p62, Loss of FOXO1/3, and Oxidative Stress Suggest the Involvement of p62-FOXO1/3 Axis in Aging

Autophagy has long been proposed and demonstrated to be involved in aging as it degrades dysfunctional organelles and macromolecules, and autophagy defect could accelerate the aging process maybe due to the accumulation of damaged organelles or molecules [53]. By far, there are many postulated theories of aging including the widespread free-radical and mitochondrial theories of aging. However, aging remains to be an intricate phenomenon with a largely elusive underlying mechanism. We detected increased protein carbonylation levels in aged rat livers compared to young ones (Figure 4(c)), demonstrating increased oxidative damage and oxidative stress in aged liver. We also observed an increased expression of p62 and NQO1 accompanied by a decreased expression of FOXO1 and FOXO3 in those aged rat livers in comparison to young ones (Figure 4(d)), indicating the inhibition of autophagy activity in aged liver. These findings suggest that the p62-FOXO1/3 axis we uncovered in the current study is involved in liver aging, and it provides us a possible interpretative perspective to the aging process which starts from declining autophagy, the driving force, to accumulation of p62, then loss of FOXO1/3, followed by impairment of antioxidant defense leading to oxidative stress and irreversible damage, eventually contributing to the progression of aging.

### 3.6. Decline of Autophagy and Accumulation of p62 but Upregulation of FOXO1/3 and Reduced ROS Level Show That p62-FOXO1/3 Axis Is Not Dominant in Type 2 Diabetes Animal Models

Autophagy was recently identified to be closely associated with obesity and type 2 diabetes by regulating lipid homeostasis and insulin sensitivity, as defective autophagy promotes ER stress, hepatic steatosis, and insulin resistance [54, 55]. We observed increased p62 along with a decreased ratio of LC3II to LC3I in the liver of ob/ob or db/db mice compared with those in the liver of c57 mice (Figures 4(e) and 4(f); [Supplementary-material supplementary-material-1]), indicating the attenuation of autophagy activity in the liver of both type 2 diabetes mice models, which could have contributed to the exacerbation of hepatic steatosis and insulin resistance. The role oxidative stress plays in type 2 diabetes could be bidirectional [56], as it is reported that increased ROS level is an important trigger for insulin resistance [57] and there is also evidence indicating the enhancement of insulin sensitivity by ROS [58]. To help disentangle the complex knot involving autophagy and ROS in type 2 diabetes, we attempted to check whether the potential p62-FOXO1/3 axis takes any effect in type 2 diabetes animal models. We found reduced ROS level but higher expression of FOXO1/3 in the liver of ob/ob or db/db mice compared with the liver of c57 mice (Figures 4(e) and 4(f); [Supplementary-material supplementary-material-1]). Consistently, it has been reported that the hyperactivation of FOXOs is associated with hyperglycemia, hypertriglyceridemia, and insulin resistance [52]. The fact that accumulated p62, upregulated FOXO1/3, and reduced ROS levels coexist in the liver of type 2 diabetes animal models indicate that the p62-FOXO1/3 axis is not dominant in diabetic liver and suggest that there are other mechanisms underlying the upregulation of FOXO1/3 in diabetic liver which could have suppressed the downregulating effect of accumulated p62 on FOXO1/3 level.

## 4. Discussion

Autophagy contributes to redox homeostasis in an antioxidative fashion not only by the clearance of oxidized cellular components as classically recognized but also by promoting antioxidant defense capacity via the p62/keap1/Nrf2 pathway in recent discoveries. In this study, we reported mitochondrial dysfunction and decrease of both expression and enzymatic activities of the antioxidant enzymes, the key components of the antioxidant defense system, in different autophagy deficiency cellular models, and revealed the p62-FOXO1/3 axis that mediates the influence of autophagy deficiency on redox balance. Our findings clearly show that inhibition of autophagy injures antioxidant enzymes which leads to oxidative stress, suggesting that autophagy deficiency impacts redox homeostasis in a prooxidant fashion by sabotaging antioxidant defense capacity besides the well-recognized insufficient clearance of oxidized cellular materials. This deepens our understanding of the regulation of redox homeostasis by autophagy to a more comprehensive sense. Furthermore, our story implicates autophagy deficiency in the etiology of aging and possibly age-related disease, too, where oxidative stress and mitochondrial dysfunction is a signature, and offers the p62-FOXO1/3 axis as potential therapeutic targeting directions in diseases involving autophagy defects.

Emerging evidences are demonstrating multifaceted roles of autophagy, especially the dynamic role of autophagy in regulating cell signaling [27, 59, 60]. p62, conventionally known as a selective autophagy receptor for the degradation of ubiquitinated substrates, has recently been proposed to function as a signaling hub for diverse cellular events [61]. Our results further corroborate the notion of p62 being an important signaling molecule as its accumulation downregulates the protein level of FOXO1/3 transcription factor that plays vital roles in coordinating metabolism and stress response in pathophysiological conditions. FOXOs serve as a converging point where numerous signaling converge and integrate to maintain homeostasis ([33, 62]). This study demonstrated the negative regulation of FOXO1/3 protein abundance by p62, indicating that FOXOs could also sense signals transduced by p62 level that integrates information derived from various cellular processes including autophagy activity. Nonetheless, the detailed molecular mechanisms by which FOXO1/3 gets downregulated in response to p62 accumulation, either through directly altering posttranslational modification of FOXO1/3 to modulating its stability or via indirect mediations of other players acting at multiple regulatory levels such as transcriptional/posttranscriptional level, warrants further investigations to elaborate. Transcriptional modulation of FOXO1/3 by p62, either directly or indirectly, should at least play a part, as mRNA levels of FOXO1/3 were lower in Atg7-/- MEF cells than in WT MEF cells ([Supplementary-material supplementary-material-1]). More interestingly, treatment with antioxidants such as N-acetyl-L-cysteine has shown benefits in circumstances related to oxidative stress or autophagy defect, like mitochondrial dysfunction and DNA damage [27], whereas NAC treatment of Atg7-/- MEFs and autophagy inhibitor-treated cells not only failed to restore protein levels of FOXO1/3 and antioxidant enzymes but even aggravated the losses. Since FOXO gene expression itself is sensitive to redox state [52], we infer that FOXO1/3 is the pivot where redox balance and autophagy activity crosstalk with each other, and the net result of FOXO1/3's integration of signals from both sources and possibly other sources orchestrate in cooperation with cofactors the expression of FOXO1/3's target genes including autophagy machinery and antioxidant enzymes. Our results imply that NAC treatment has a dark side as a therapeutic approach because quenching ROS by NAC might downregulate the FOXO-antioxidant pathway and most likely autophagy activity too, which could in turn weaken the endogenous cellular antioxidant capacity. Therefore, pharmacological approaches activating FOXO activity might be promising strategies for treating diseases induced by autophagy defects.

The predominant role of FOXOs is to respond to and counteract stress conditions for the maintenance of cellular homeostasis, and it can be activated by metabolic and oxidative stress [33]. Autophagy-deficient cells are known to suffer from severe oxidative stress and DNA damage due to the well-characterized defects in the clearance of damaged and aberrant organelles such as mitochondria, and accumulation of dysfunctional mitochondria due to absent autophagy results in the generation of more reactive species that would further harm mitochondria, feeding a vicious cycle [27]. Our results show that FOXOs fail to respond to the enhanced oxidative stress present in an autophagy deficiency condition by upregulating antioxidant enzymes as the accumulation of p62 negatively regulates the protein levels of FOXO1/3, suggesting that autophagy deficiency represents a severe disturbance of homeostasis where stress response depending on FOXO1/3 is wrecked and homeostasis can no longer be recovered. Therefore, autophagy-deficient cells are much more vulnerable to cellular oxidative damage, implying that enhancing autophagy could have therapeutic potential in oxidative stress-associated pathologies and increasing antioxidant power could render benefits for diseases implicating autophagy deficiency. Indeed, increasing basal autophagy levels could extend lifespan and enhance resistance to oxidative stress in adult *Drosophila* [63] and in mice [64]; treatment with antioxidants rescued the defects of intestinal stem cell-dependent intestinal recovery after irradiation in mice lacking Atg5 [65], and NAC treatment ameliorated the impairment of glucose tolerance in pancreatic *β* cells in autophagy-deficient models by reducing continuous oxidative stress ([29]).

The probable cause-and-effect relationship between autophagy and aging has been investigated and discussed intensively in the past decade. Generally, autophagy diminishes with both normal and pathological aging, autophagy inhibition leads to premature aging, and autophagy defect accelerates the aging process while stimulating autophagy delays aging and extends longevity [53]. We infer from our observations with aged and young rat liver and cellular studies that autophagy activity abates while oxidative damage heightens during rat liver aging, and the p62-FOXO1/3 axis regulating antioxidant defense might play an important role in liver aging process. Specifically, FOXO1/3 loss and the impaired antioxidant enzymes might accelerate aging or increase incidence of age-associated diseases in a condition of autophagy deficiency; therefore, targeting p62, FOXOs, or antioxidant defense might be considered in developing potential therapeutic strategies for diseases implicated with autophagy defects.

In addition, we also detected increased expression of NQO1, one of the phase II detoxifying enzymes, in aged rat liver, and increased expression of NQO1 as well as its upstream regulator Nrf2 in Atg7-/- MEF cells (Figure 4(d); Figures [Supplementary-material supplementary-material-1] and [Supplementary-material supplementary-material-1]). NQO1 is a highly inducible detoxification enzyme regulated by the Keap1/Nrf2/ARE pathway; it promotes 2/4-electron reductions of quinones and can minimize reactive oxygen intermediate generation from redox cycling and reserve intracellular thiol pools; its important antioxidant function in combating oxidative stress has been well substantiated [66]. Our results are consistent with previous findings that autophagy-deficient models exhibit hyperactivated Nrf2 transcription activity and enhanced induction of Nrf2 target genes such as NQO1, possibly due to the competitive inhibition of Nrf2-Keap1 interactions by accumulated p62 which can bind to Keap1 and mediate its degradation [32]. These findings suggest that upregulation or activation of the Nrf2/ARE/NQO1 pathway might occur in aged liver, which the abated autophagy activity in aged liver is probably partly accountable for. Meanwhile, the overall increase of oxidative damage in aged rat liver implies that the activation of the Nrf2/ARE/NQO1 pathway is not sufficient to confer enough antioxidant capacity to protect against oxidative stress induced by autophagy decline during aging. The downregulation of more vital antioxidant enzymes like Cu-ZnSOD, MnSOD, and catalase by the p62-FOXO1/3 axis is probably more decisive in overall output of antioxidant defense capacity in aged rat liver. As can be seen, the regulation of antioxidant enzymes during aging involves versatile transcription factors like Nrf2 and FOXO1/3, which integrates a wide spectrum of upstream signals including information delivered by p62 which together produce the actual net outcome. In addition, NQO1's cytoprotective roles unrelated to enzymatic activities have been demonstrated; for instance, it selectively binds to and hence stabilizes specific proteins like p53, a well-known tumor suppressor, against proteasomal degradation [66]. This suggests that the increase of NQO1 expression in aged rat liver might have other significances beyond its antioxidant function, which might involve regulation of the degradative fate of certain important proteins, and contribute to the prevention and protection against tumorigenesis during aging. Further studies are warranted to fully decipher the roles of NQO1 in aging.

Autophagy plays intricate roles in type 2 diabetes. There are paradoxical reports that autophagy defect promotes insulin resistance, the core of type 2 diabetes ([55]), and autophagy deficiency leads to protection from obesity and insulin resistance by inducing mitokine [67]. Taken together with our observations, we can see that the driving force is derived from the lack of leptin or leptin receptor as in ob/ob or db/db mice; the attenuation of autophagy activity eventually occurs and could further contribute to the progression of type 2 diabetes or act as a protective response against the exacerbation of insulin resistance. FOXO proteins are crucial in diabetes as they are highly expressed in the major insulin target tissues and play multiple and complex roles as both overexpression of constitutively active FOXO1*α* and knockdown of FOXO1/3*α* could lead to diabetes or hypertriglyceridemia [52]. In our results, FOXO1/3 exhibits higher expression levels in the liver of ob/ob or db/db mice in comparison to those of c57 mice. FOXOs also have beneficial effects in the context of diabetes, as FOXO-dependent transcription of antioxidant enzymes may counteract oxidative stress-induced cellular damage [52], which agrees with our findings that overall ROS level is lower in liver homogenates of db/db mice compared to those of c57, though expression of Cu-ZnSOD and catalase in the liver of ob/ob or db/db mice is similar to that of c57 mice. These findings suggest that the p62 accumulation due to diminished autophagy in diabetic liver was not able to decrease FOXO1/3 which is hyperactivated by other mechanisms and yields an overall decrease in ROS level despite that hyperglycemia typically induces oxidative stress. In fact, the relationship between autophagy and antioxidant defense is neither simple nor straightforward, depending on specific contexts. A study of the intestinal epithelial cell-specific Atg5 knockout mice model showed that activation of the ERK/Nrf2/HO-1 pathway by oxidative stress in a condition of autophagy deficiency led to diminished indomethacin-induced intestinal epithelial cell damage because of the stronger resistance to oxidative stress [68]. Yu et al. [69] demonstrated that catalase could be selectively degraded by autophagy in caspase inhibition conditions, thus leading to programmed cell death induced by abnormal ROS accumulation. To fully unravel the interplay of autophagy, FOXOs, and redox balance in a diabetic setting and lay groundwork for potential therapeutic strategies, further studies are called to dissect more molecular events.

In conclusion, the present study demonstrates that inhibition of autophagy by either chemical or genetic approaches leads to mitochondrial dysfunction, oxidative stress, and impaired expressions and enzymatic activities of the antioxidant enzymes; the p62-FOXO1/3 axis underlies the impairment of antioxidant defense in an autophagy deficiency condition. We propose that autophagy deficiency could disrupt intracellular homeostasis through injuring the antioxidant system besides the incapability of clearing damaged components; and accumulation of damaged mitochondria and the impaired antioxidant defense enhance oxidative stress together in a condition of autophagy deficiency. The newly uncovered p62-FOXO1/3 axis is probably linking autophagy decline to oxidative stress in liver aging, which provides a perspective to help reveal the mystery of aging. Hopefully, our study could shed light on the path elucidating the interplay between autophagy and oxidative stress and the quest for the mechanisms of aging and pathophysiology of autophagy-defect-related diseases, and could lay ground for potential therapeutic strategies.

## Data Availability

The biochemical data, gene expression data, and Western blot data used to support the findings of this study are included within the article and the supplementary figures. Any additional data used to support the findings of this study are available upon request.

## Additional Points

*Highlights*. (i) Inhibition of autophagy by chemical inhibitors, knockdown of Atg5 or Beclin-1, or Atg7 knockout leads to mitochondrial dysfunction, elevated ROS level, impaired antioxidant defense, and downregulated FOXO1/3 transcription factors. (ii) Overexpression of FOXO1/3 rescues the impairment of antioxidant enzymes and reduces the elevation of ROS level under an autophagy deficiency condition. (iii) p62 knockdown increases the expression of FOXO1/3, and p62 knockdown reverses the downregulation of FOXO1 in a condition of Atg5 knockdown where p62 accumulates. (iv) Accumulated p62, loss of FOXO1/3, and increased oxidative damage occur in aged rat liver, whereas accumulated p62, upregulated FOXO1/3, and reduced ROS level coexist in the liver of a type 2 diabetes mice model, suggesting that the p62-FOXO1/3 axis is involved in liver aging but not dominant in diabetic liver

## Supplementary Materials

Supplementary Materials Figure S1: CQ treatment results in decreased mRNA levels of autophagy components in WT MEF cells in a dose-dependent manner, and mRNA levels of autophagy components decrease in Atg7-/- MEF cells compared with WT. Figure S2: Nrf2 and NQO1 expression is upregulated in Atg7-/- MEF cells compared with WT. Figure S3: overexpression of FOXOs restores mRNA levels of antioxidant enzymes as well as autophagy components. Figure S4: expression levels of indicated proteins in the liver tissues of 18-week-old ob/ob (B6/JNju-Lepem1Cd25/Nju), db/db (BSK-Leprem2Cd479 889/Nju), and C57 mice were determined by W.B. (*n* = 6).
