# Supplementary material for: Autophagy Deficiency Leads to Impaired Antioxidant Defense via p62-FOXO1/3 Axis
Source: Oxid Med Cell Longev. 2019 Dec 17;2019:2526314. doi: 10.1155/2019/2526314 (PMC6935825; doi:10.1155/2019/2526314)
Supplement: Supplementary Materials — Figure S1: CQ treatment results in decreased mRNA levels of autophagy components in WT MEF cells in a dose-dependent manner, and mRNA levels of autophagy components decrease in Atg7-/- MEF cells compared with WT. Figure S2: Nrf2 and NQO1 expression is upregulated in Atg7-/- MEF cells compared with WT. Figure S3: overexpression of FOXOs restores mRNA levels of antioxidant enzymes as well as autophagy components. Figure S4: expression levels of indicated proteins in the liver tissues of 18-week-old ob/ob (B6/JNju-Lepem1Cd25/Nju), db/db (BSK-Leprem2Cd479 889/Nju), and C57 mice were determined by W.B. (n = 6). [file 2526314.f1.pptx]

## Slide 1
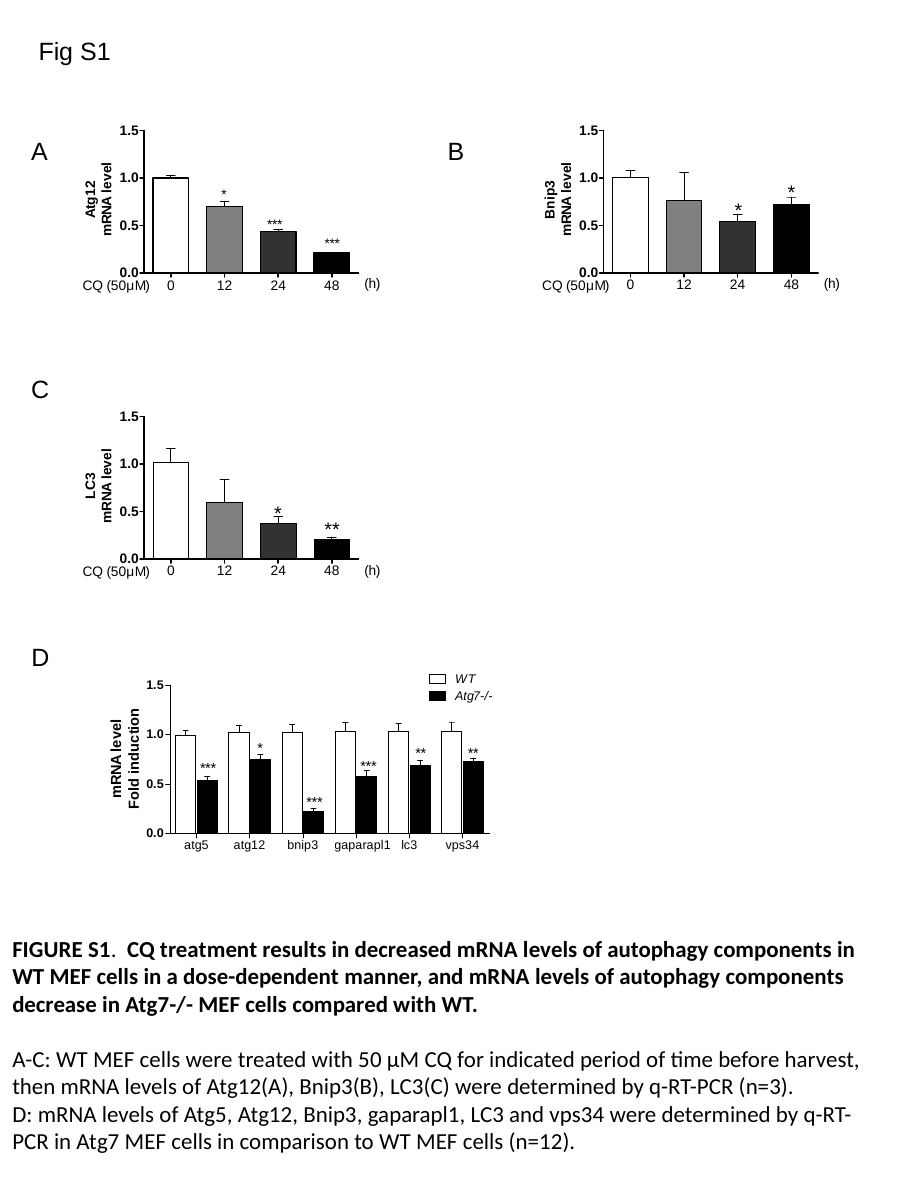

Fig S1
A
B
C
D
FIGURE S1. CQ treatment results in decreased mRNA levels of autophagy components in WT MEF cells in a dose-dependent manner, and mRNA levels of autophagy components decrease in Atg7-/- MEF cells compared with WT.
A-C: WT MEF cells were treated with 50 µM CQ for indicated period of time before harvest, then mRNA levels of Atg12(A), Bnip3(B), LC3(C) were determined by q-RT-PCR (n=3).
D: mRNA levels of Atg5, Atg12, Bnip3, gaparapl1, LC3 and vps34 were determined by q-RT-PCR in Atg7 MEF cells in comparison to WT MEF cells (n=12).

## Slide 2
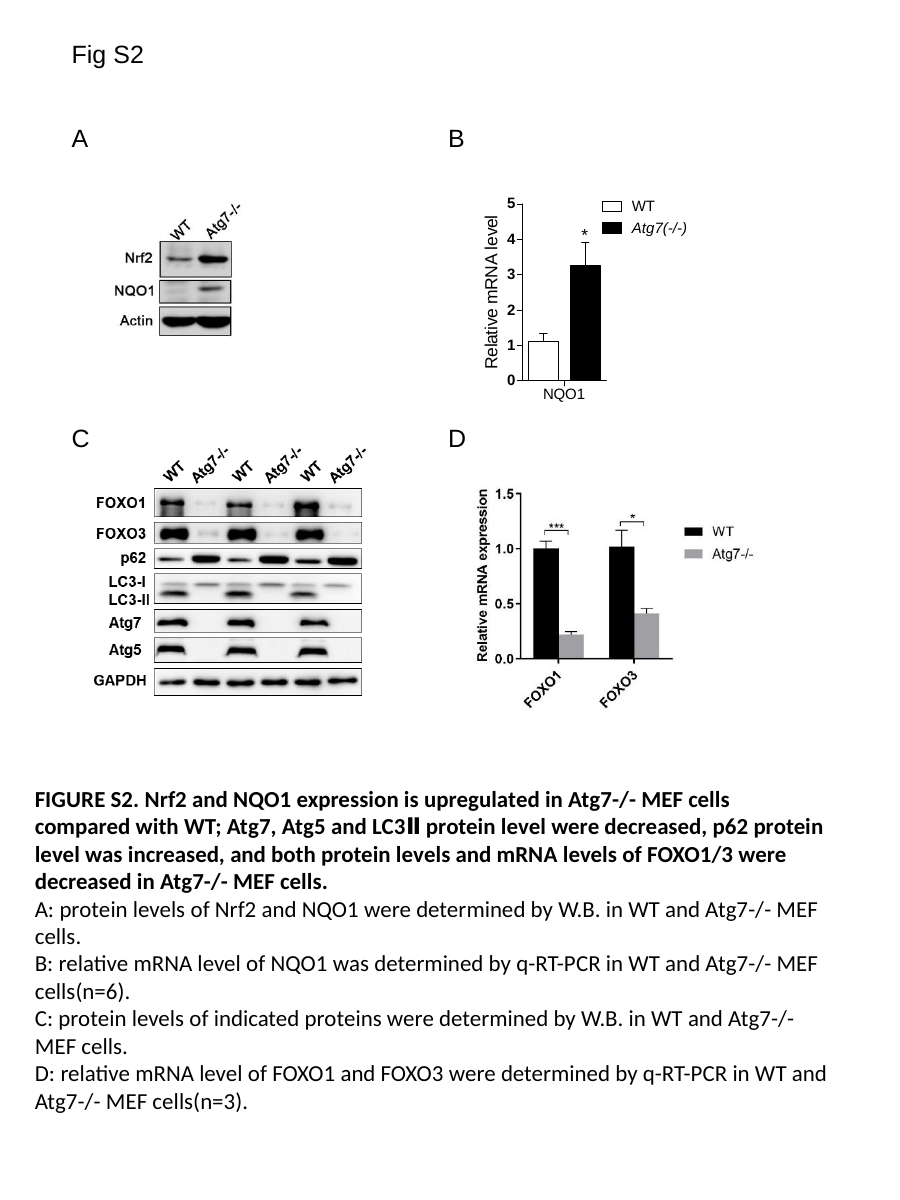

Fig S2
A
B
D
C
FIGURE S2. Nrf2 and NQO1 expression is upregulated in Atg7-/- MEF cells compared with WT; Atg7, Atg5 and LC3Ⅱ protein level were decreased, p62 protein level was increased, and both protein levels and mRNA levels of FOXO1/3 were decreased in Atg7-/- MEF cells.
A: protein levels of Nrf2 and NQO1 were determined by W.B. in WT and Atg7-/- MEF cells.
B: relative mRNA level of NQO1 was determined by q-RT-PCR in WT and Atg7-/- MEF cells(n=6).
C: protein levels of indicated proteins were determined by W.B. in WT and Atg7-/- MEF cells.
D: relative mRNA level of FOXO1 and FOXO3 were determined by q-RT-PCR in WT and Atg7-/- MEF cells(n=3).

## Slide 3
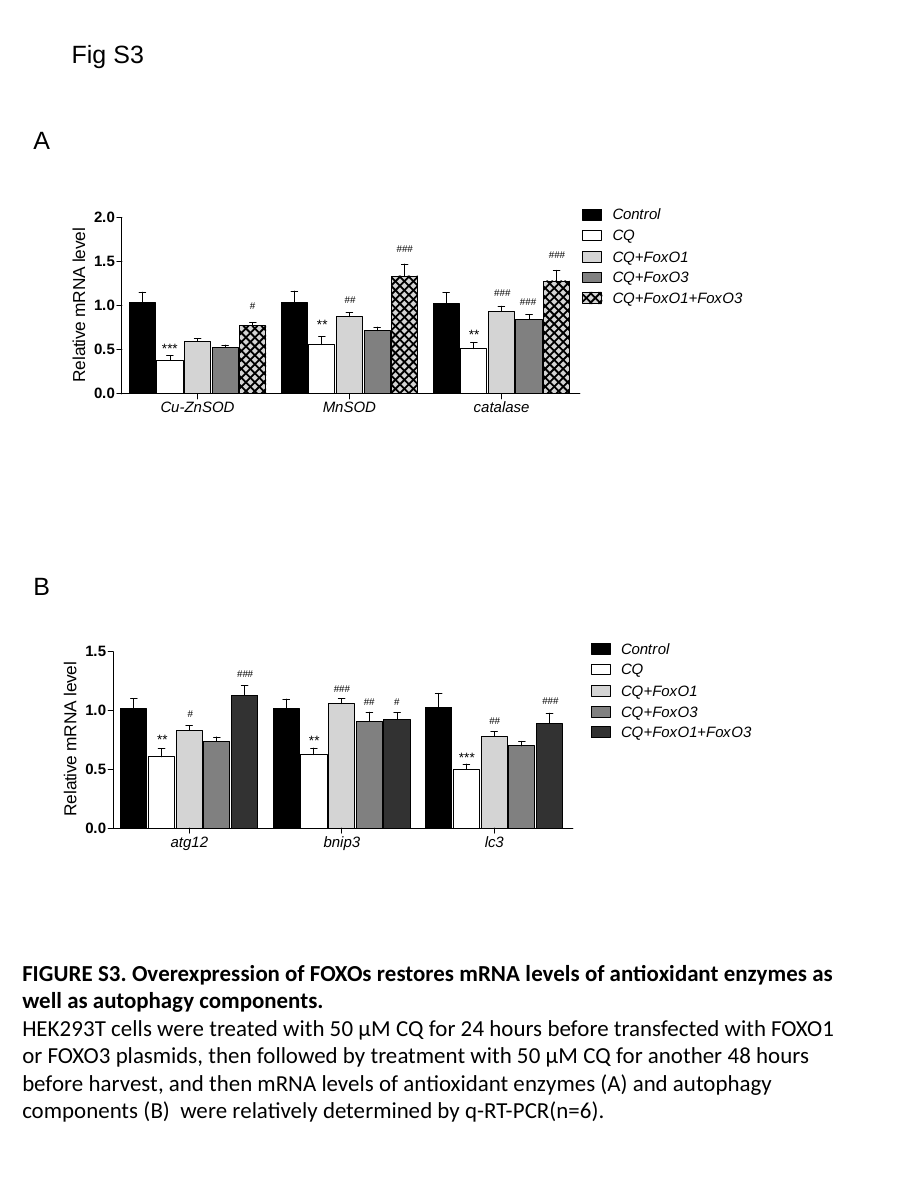

Fig S3
A
B
FIGURE S3. Overexpression of FOXOs restores mRNA levels of antioxidant enzymes as well as autophagy components.
HEK293T cells were treated with 50 µM CQ for 24 hours before transfected with FOXO1 or FOXO3 plasmids, then followed by treatment with 50 µM CQ for another 48 hours before harvest, and then mRNA levels of antioxidant enzymes (A) and autophagy components (B) were relatively determined by q-RT-PCR(n=6).

## Slide 4
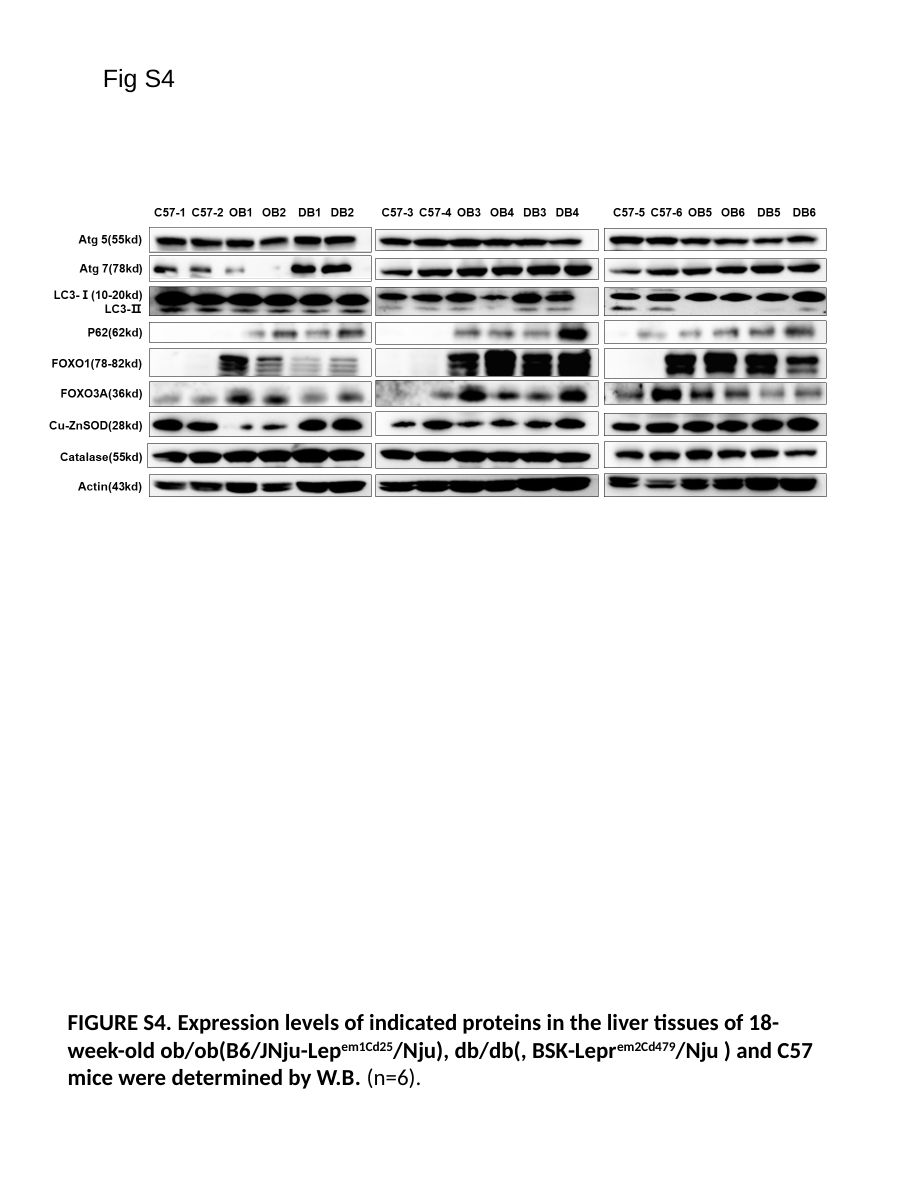

Fig S4
FIGURE S4. Expression levels of indicated proteins in the liver tissues of 18-week-old ob/ob(B6/JNju-Lepem1Cd25/Nju), db/db(, BSK-Leprem2Cd479/Nju ) and C57 mice were determined by W.B. (n=6).
